# AI‐Enabled Next‐Generation Dairy Systems: From Sensors to Smart Processing

**DOI:** 10.1002/fsn3.71953

**Published:** 2026-05-27

**Authors:** Zeki Erol, Jerina Rugji, Ambreen Hamadani, Henna Hamadani

**Affiliations:** ^1^ Department of Food Hygiene and Technology, Faculty of Veterinary Medicine Dokuz Eylul University Izmir Turkey; ^2^ Independent Researcher Izmir Turkey; ^3^ Sher‐e‐Kashmir University of Agricultural Sciences and Technology of Kashmir Kashmir India

**Keywords:** artificial intelligence, Dairy 4.0, milk quality assessment, precision livestock farming

## Abstract

The rapid integration of artificial intelligence (AI) into dairy systems has been widely promoted under the Dairy 4.0 paradigm, yet its role as a true system‐level “game changer” remains insufficiently substantiated. This study presents a structured critical review of AI‐enabled technologies across the dairy value chain, encompassing precision livestock farming, in‐line milk quality sensing, smart processing, and advanced ingredient development. A transparent literature search and selection strategy was applied to identify and evaluate relevant studies, with emphasis on reported performance metrics, validation conditions, and real‐world applicability. The analysis reveals that while AI demonstrates strong technical capability, particularly in milk quality prediction, sensor‐based monitoring, and process optimization, most evidence is derived from controlled laboratory settings, with limited validation under heterogeneous farm and industrial conditions. Key limitations include challenges in model generalizability, data integration, calibration stability, and interoperability with existing infrastructure. Moreover, economic feasibility, scalability across production systems, and governance issues related to data ownership and accountability remain insufficiently addressed. Across the reviewed domains, the impact of AI is found to be conditional rather than inherently transformative. Technologies deliver measurable benefits primarily when embedded within sensor‐rich, interoperable systems that directly inform operational decision‐making. In contrast, standalone AI applications often function as analytical tools without inducing system‐level change. The review further highlights discrepancies between technological potential and demonstrated outcomes, particularly in sustainability performance and industrial‐scale implementation. Overall, AI should be conceptualized not as an autonomous disruptive force but as a system‐level enabler, whose effectiveness depends on complementary advances in sensing, infrastructure, data governance, and workforce capability. Future research should prioritize field validation, economic assessment, and integration frameworks to bridge the gap between experimental performance and practical deployment, thereby enabling more realistic evaluation of AI's transformative potential in dairy systems.

## Graphical abstract

AI‐enabled Dairy 4.0 technologies integrate big data, IoT, smart sensing, digital twins, and machine learning to address key dairy industry challenges including sustainability, milk quality variability, animal health, traceability, and resource efficiency. These system‐level AI applications support precision livestock farming, intelligent dairy processing, and sustainable bioeconomy strategies by enabling real‐time monitoring, predictive optimization, and reduced environmental impact.

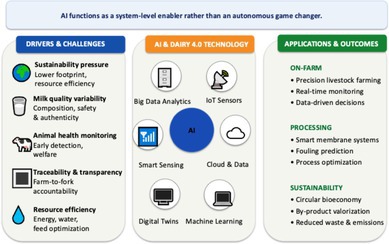

## 1 Introduction

Global food systems face a dual burden of overnutrition and persistent micronutrient deficiency, placing unprecedented pressure on food production to deliver higher nutritional value with lower environmental impact. Recent estimates from the World Health Organization indicate that obesity affects billions worldwide, while hidden hunger remains widespread (World Health Organization 2025). Within this context, dairy systems occupy a pivotal yet contested role. Milk provides high‐quality protein and essential micronutrients and is increasingly positioned as a model matrix for nutrition and health research (German et al. 2022). However, its environmental footprint, inherent biological variability, and processing inefficiencies complicate its contribution to sustainable food systems (Bhat and Infascelli 2025; Cruz‐Rivero et al. 2025).

The sector is responding through rapid digitalization under the Dairy 4.0 paradigm, integrating sensors, Internet of Things technologies, automation, and AI across production and processing (Hassoun et al. 2023; Espinoza‐Sandoval et al. 2024). Precision livestock farming enables continuous monitoring of animal health, behavior, and milk quality, while advanced processing technologies, including in‐line sensing and membrane systems, aim to improve efficiency and reduce losses (Knight 2020; Reig et al. 2021; Zhang et al. 2023). Despite this momentum, claims that AI represents a “game changer” remain premature. Transformative innovation requires not only improved performance but a fundamental shift from reactive, fragmented management toward predictive, integrated, and scalable decision‐making (Avelino et al. 2017). To date, most studies demonstrate technical feasibility under controlled conditions, with limited evidence for field validation, economic viability, and long‐term adoption.

This gap is particularly evident in milk quality assessment. Machine learning models combined with spectroscopic tools report high accuracy in detecting adulteration and predicting composition (Chaudhary et al. 2022; Chu et al. 2024; Aqeel et al. 2025). However, laboratory performance rarely translates directly to commercial environments, where variability in breed, feeding, lactation stage, and operational conditions challenge model robustness. Even established methods such as near‐infrared spectroscopy remain highly sensitive to calibration quality, temperature effects, and instrument transferability (Surkova et al. 2019; Díaz‐Olivares, Gote, et al. 2024; Díaz‐Olivares, Grauwels, et al. 2024). The critical challenge, therefore, is not accuracy per se, but robustness, interpretability, and decision relevance across heterogeneous dairy systems.

Similar limitations arise in biosensing and animal health monitoring. Sensors targeting mastitis‐related indicators enable early detection, yet their performance is constrained by false positives, signal drift, and limited specificity when deployed in isolation (Norberg et al. 2004; Martins et al. 2019; Pan et al. 2025). While multi‐sensor integration and AI can enhance predictive capacity, they also increase reliance on data quality, interoperability, and user trust. Accordingly, the value of AI‐enabled monitoring should be assessed by its ability to improve actionable decisions, such as treatment, milk segregation, and herd management, rather than predictive metrics alone.

At the processing level, AI‐assisted membrane systems and digital twins are frequently presented as transformative. Predictive fouling models may enable optimized cleaning and energy efficiency, while digital twins offer potential for simulation and adaptive control (Niu et al. 2022; Zhang et al. 2023). Yet their practical impact depends on sufficient sensor infrastructure, automation, and control authority within processing plants. Industrial validation in dairy remains limited, and claims of transformation are often disproportionate to demonstrated real‐world performance.

Beyond technical performance, AI adoption is fundamentally socio‐technical. Digital dairy systems require investment, infrastructure, and data literacy, creating uneven adoption trajectories that may favor large‐scale operations unless inclusive models are developed (Krampe et al. 2024; Hostens et al. 2025). Concurrently, unresolved challenges related to data ownership, privacy, standardization, and algorithmic accountability constrain system‐wide integration (Malik et al. 2024a, 2024b). These factors will ultimately determine whether AI enables inclusive transformation or reinforces existing inequalities.

Supply‐chain integration further complicates this landscape. While precision livestock farming and optimization models offer pathways to improved efficiency and sustainability, their implementation remains limited by data fragmentation and infrastructure constraints (Lovarelli et al. 2020; Malik et al. 2022). Traceability systems, including blockchain‐based approaches, have been proposed to enhance transparency, yet their effectiveness depends on reliable data and stakeholder alignment (Hastig and Sodhi 2020). A critical bottleneck remains the lack of interoperability across digital platforms, which restricts coordinated decision‐making and scalability (Malik, Gahlawat, et al. 2023; Malik, Malik, et al. 2023).

Collectively, the evidence indicates that AI holds substantial promise for dairy systems, but its transformative potential remains conditional. Current research is strong in demonstrating technical capability but weak in external validation, scalability, economic return, and governance readiness. This review therefore critically examines AI‐enabled dairy systems across the value chain, focusing on the gap between laboratory performance and field‐level impact, the conditions required for scalable implementation, and the extent to which AI can function as a true system‐level innovation. Ethical considerations, including animal welfare and agency, are integral to this assessment, ensuring that technological progress does not reduce animals to mere data sources (Kramer and Bovenkerk 2024). Recent literature suggests that the transition toward AI‐enabled dairy systems should be interpreted through broader digital transformation and socio‐technical perspectives rather than through a purely technological lens. Industry 4.0 integration in dairy systems depends not only on algorithmic capability, but also on organizational readiness, infrastructure maturity, interoperability, governance structures, and stakeholder adoption (Malik, Gahlawat, et al. 2023; Hassoun et al. 2023). In this context, the Technology‐Organization‐Environment (TOE) framework provides a useful conceptual basis for understanding dairy digitalization by linking technological readiness, organizational capabilities, and external environmental pressures, including sustainability regulations, traceability demands, and market competitiveness. Simultaneously, socio‐technical systems (STS) theory emphasizes that AI‐driven systems operate through interactions among sensors, operators, farmers, animals, processing infrastructure, and institutional conditions, rather than functioning as isolated analytical tools. This interpretation aligns with digital transformation theory, in which transformation is defined not merely by technology adoption, but by the restructuring of operational processes, decision‐making systems, and value‐chain interactions. Accordingly, the transformative potential of AI in dairy systems should be considered conditional and context‐dependent, requiring integration across technological, organizational, economic, and social dimensions before meaningful system‐level change can occur (Malik et al. 2024a; Lovarelli et al. 2020; Hastig and Sodhi 2020). Furthermore, recent studies emphasize that the absence of interoperability, standardization, and governance frameworks remains a major bottleneck preventing scalable implementation of Dairy 4.0 systems across heterogeneous production environments (Malik, Gahlawat, et al. 2023; Malik, Malik, et al. 2023).

## 2 Methodology

**FIGURE 1:**
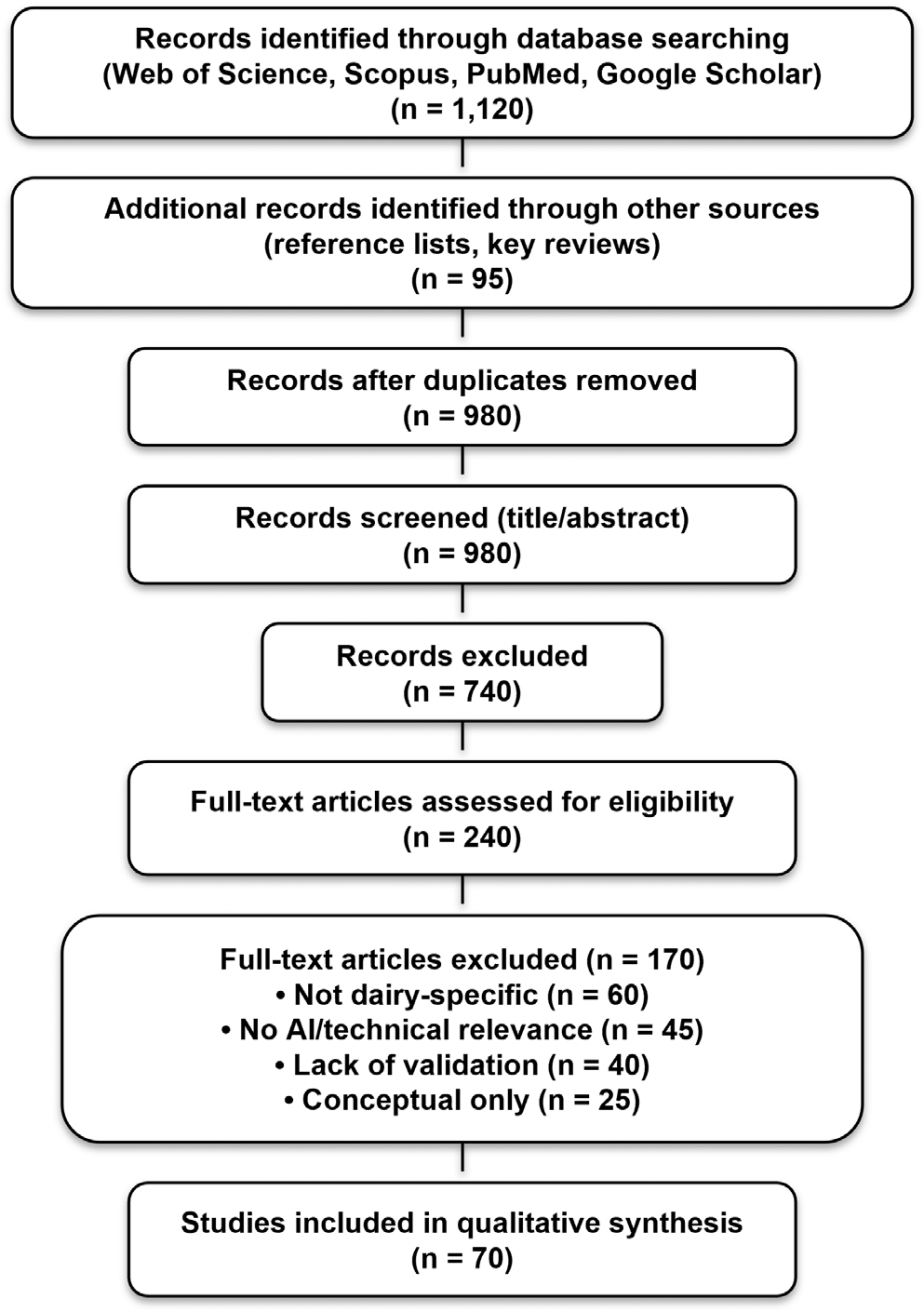
PRISMA‐informed flow diagram showing the literature identification and screening process used for this structured critical review.

This study was conducted as a structured critical review to evaluate the role of AI and advanced digital technologies across the dairy value chain, including precision livestock farming, milk quality assessment, smart sensing, traceability, digital twins, sustainability, and next‐generation dairy processing systems. The methodological design was informed by PRISMA 2020 reporting principles and recent systematic literature review (SLR) approaches applied in Dairy 4.0 and Industry 4.0 research (Page et al. 2021; Malik et al. 2022; Malik, Gahlawat, et al. 2023). A systematic literature search was conducted using Web of Science, Scopus, PubMed, and Google Scholar to identify peer‐reviewed studies published between 2000 and January 2026, with emphasis on studies published after 2015 to capture recent developments associated with Dairy 4.0 technologies. Search queries combined keywords including “artificial intelligence,” “machine learning,” “dairy,” “milk,” “sensor,” “biosensor,” “NIR spectroscopy,” “precision livestock farming,” “digital twin,” “traceability,” “Industry 4.0,” and “membrane processing,” using Boolean operators (AND/OR) to refine search outputs. Reference lists of key review papers were additionally screened to identify relevant studies not captured during the primary database search. The review process followed a PRISMA‐informed workflow involving database identification, duplicate removal, title and abstract screening, eligibility assessment, and full‐text evaluation (Figure 1). Studies were included if they: (i) were peer‐reviewed, (ii) focused on AI‐enabled or advanced digital technologies in dairy systems, and (iii) reported quantitative performance metrics, validation outcomes, or experimentally supported findings. Studies were excluded if they: (i) were unrelated to dairy systems, (ii) lacked methodological transparency, or (iii) consisted solely of conceptual discussion without empirical or analytical support. Data extracted from eligible studies included application domain, AI or machine‐learning method, sensing or processing technology, dataset characteristics, validation setting, reported performance indicators, implementation scale, interoperability considerations, operational limitations, economic feasibility, and sustainability relevance. Studies were subsequently categorized into thematic domains, including precision livestock farming, milk quality assessment, smart processing technologies, digital traceability, supply‐chain optimization, sustainability assessment, and circular bioeconomy integration. Because substantial heterogeneity existed across datasets, technologies, experimental conditions, and evaluation methods, a structured critical synthesis approach was adopted instead of meta‐analysis. This approach enabled comparative evaluation of technical performance, robustness, scalability, operational feasibility, economic practicality, interoperability, and system‐level impact across different dairy applications. To improve analytical consistency, a multi‐dimensional evaluation framework was applied to classify technologies according to: (1) technical performance, (2) robustness and generalizability, (3) operational feasibility, (4) scalability and economic considerations, and (5) transformative potential within dairy systems. Based on these criteria, AI applications were critically interpreted as incremental, enabling, or potentially transformative technologies rather than assuming that digitalization alone guarantees system‐level innovation. The methodological structure additionally aligns with recent SLR‐based studies addressing digital transformation, interoperability, traceability, and optimization frameworks in dairy systems (Malik et al. 2022; Malik, Gahlawat, et al. 2023; Malik, Malik, et al. 2023).

## 3 Results and Discussion

### 3.1 Next‐Generation Dairy Systems: Concept, Drivers, and Global Trends

**FIGURE 2:**
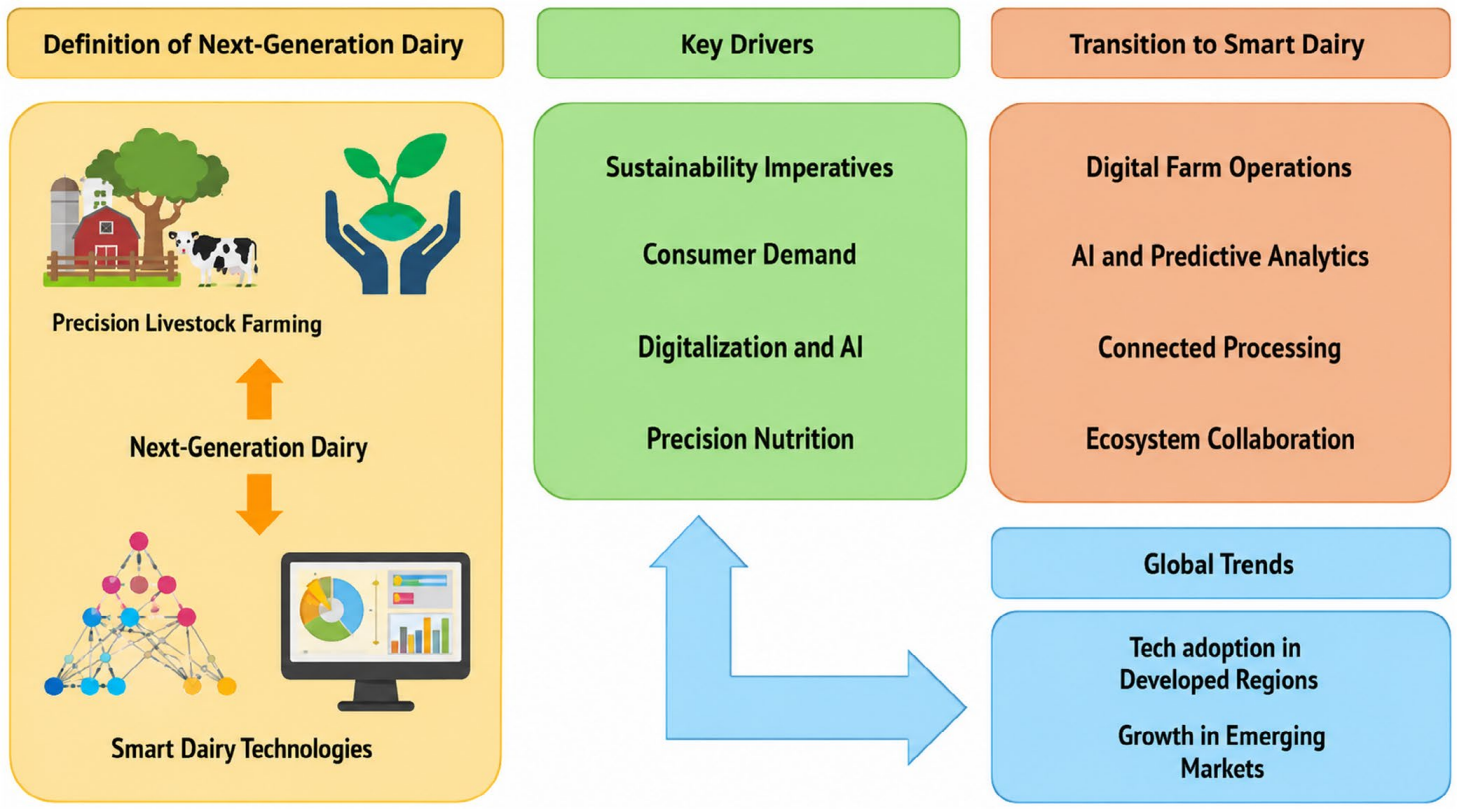
TOE‐informed conceptual framework of next‐generation dairy systems: Technological, organizational, and environmental drivers of Dairy 4.0 adoption. *Source:* The figure was developed by the authors for this manuscript.

The literature positions next‐generation dairy systems as a transition from labor‐intensive, episodic decision‐making toward data‐driven, interconnected, and increasingly autonomous production systems. This transformation spans the entire value chain, integrating on‐farm monitoring, smart raw milk management, adaptive processing, and advanced ingredient innovation. Precision Livestock Farming (PLF), enabled by IoT sensors and machine learning, is widely recognized as a foundational component of this transition, allowing continuous monitoring of animal health, behavior, and productivity (Trapanese et al. 2025). In parallel, Dairy 4.0 technologies—such as robotics, automation, and digital control systems—are reported to enhance processing efficiency and consistency (Hassoun et al. 2023). However, a critical synthesis of the literature indicates that these developments are often framed in aspirational terms, with limited empirical validation at scale. While technical feasibility is well established, the translation of these technologies into measurable improvements in sustainability, economic performance, or system resilience remains inconsistent. Many studies implicitly assume that digitalization leads to optimization, yet evidence demonstrating sustained, system‐wide benefits across diverse production contexts is scarce. The drivers of this transition, sustainability pressures, consumer demand for traceability and animal welfare, and the need for efficiency, are widely acknowledged (Priyashantha 2025; de Oliveira et al. 2024). Nevertheless, their interaction is rarely critically examined. For example, while digital monitoring is frequently linked to environmental mitigation, quantitative evidence of reduced emissions or resource use attributable to AI‐enabled systems is limited. Similarly, traceability technologies are often presented as solutions for transparency, yet their effectiveness depends on data standardization, governance, and stakeholder alignment, which remain unresolved challenges. Adoption patterns further reveal structural inequalities. Advanced digital systems are concentrated in high‐income regions with strong infrastructure and financial capacity, whereas implementation in emerging systems depends heavily on training, service models, and institutional support (Trapanese et al. 2025; de Oliveira et al. 2024). This uneven distribution suggests that AI and digitalization are context‐dependent innovations rather than universally scalable solutions. Consequently, the transition toward next‐generation dairy systems should be interpreted as a gradual, nonlinear process, rather than a uniform technological shift. Figure 2 can be interpreted through the Technology‐Organization‐Environment framework, which explains how technological innovation in dairy systems emerges from interactions among technical capability, organizational readiness, and external environmental pressures. Technological dimensions include AI algorithms, IoT‐based sensing, automation, digital twins, and interoperability platforms, while organizational dimensions encompass workforce capability, investment readiness, management support, and operational integration. Environmental dimensions include sustainability pressures, food safety requirements, traceability regulations, consumer expectations, and supply‐chain competitiveness. This integrated interpretation highlights that digital transformation in dairy systems is not solely technology‐driven but depends on coordinated adaptation across technical, institutional, and economic domains. Similar conclusions have been reported in recent Industry 4.0 and dairy digitalization studies, which emphasize that infrastructure limitations, fragmented data ecosystems, and lack of interoperability remain major barriers to widespread implementation (Malik, Gahlawat, et al. 2023; Malik et al. 2024a; Hassoun et al. 2023).

### 3.2 Smart Raw Milk Management and Quality Assessment

#### 3.2.1 In‐Line Sensing and Data Integration

**FIGURE 3:**
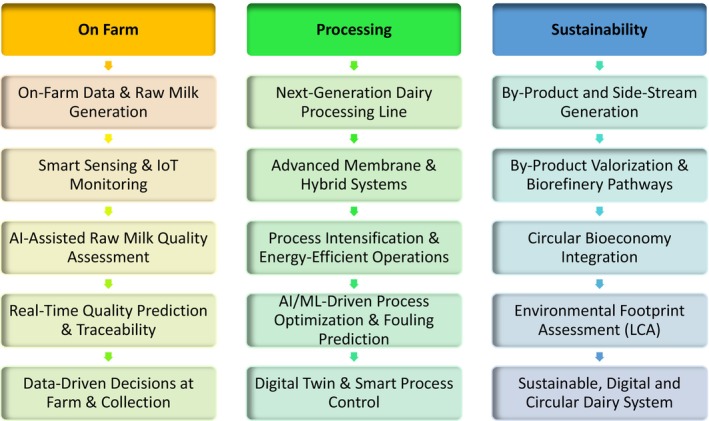
Socio‐technical and digital transformation framework for AI‐enabled next‐generation dairy systems. *Source:* The figure was developed by the authors for this manuscript.

**FIGURE 4:**
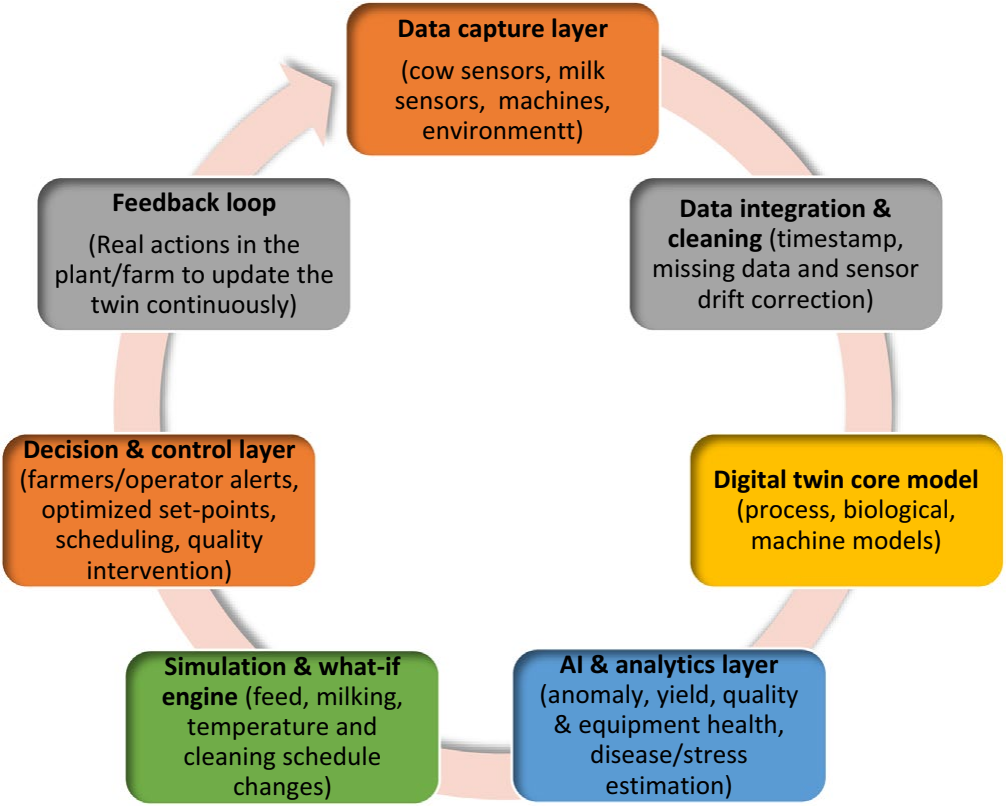
Digital twin workflow for the dairy industry. *Source:* The figure was developed by the authors for this manuscript.

Raw milk management represents a critical entry point for digital transformation due to its direct impact on product quality, safety, and processing performance. Traditional laboratory‐based testing, although accurate, is limited by temporal delays. In contrast, in‐line sensing technologies enable continuous monitoring and earlier intervention, potentially reducing variability and losses. Despite this potential, the effectiveness of in‐line sensing is highly dependent on data integration and decision‐making frameworks. The “at cow,” “near cow,” and “from cow” data paradigm (Knight 2020) highlights the increasing complexity of data streams, but also underscores challenges related to interoperability, data quality, and system integration. Many studies demonstrate improved detection capabilities, yet few evaluate how these data translate into actionable and economically meaningful decisions at the farm level. The integrated framework presented in Figure 3 also reflects a socio‐technical systems perspective in which dairy digitalization is shaped by interactions among sensing technologies, AI‐assisted analytics, farm operators, processing infrastructure, governance systems, and sustainability objectives. Within this framework, real‐time monitoring, smart sensing, AI‐driven prediction, and digital‐twin applications function as interconnected decision‐support mechanisms rather than isolated technologies. The framework additionally aligns with broader digital transformation concepts by integrating operational efficiency, circular bioeconomy principles, traceability, environmental assessment, and process optimization into a unified adaptive system. Such integration is increasingly recognized as essential for achieving scalable and sustainable Dairy 4.0 implementation because technological performance alone does not guarantee practical transformation without interoperability, stakeholder coordination, and reliable data ecosystems (Hassoun et al. 2023; Malik, Malik, et al. 2023). Figure 3 presents a conceptual framework integrating the key drivers, mechanisms, and outcomes associated with AI‐enabled dairy systems. At the driver level, increasing demands for nutritional quality, sustainability, and system efficiency create pressure for innovation. These drivers are operationalized through underlying mechanisms, including data acquisition, machine learning‐based modeling, and process optimization, which together enable the translation of complex dairy system data into actionable insights. The resulting outcomes include improved product functionality, enhanced process control, and greater resource efficiency. Importantly, feedback loops between outcomes and system inputs highlight the adaptive and iterative nature of these systems. Figure 4 represents a digital twin workflow for the dairy industry. Figure 4 synthesizes the interactions between system components by illustrating how data inputs, modeling approaches, and operational constraints converge to influence performance outcomes. The figure highlights that system effectiveness is not determined solely by algorithmic accuracy, but by the alignment between data quality, model design, and implementation context. Importantly, it emphasizes that variability in real‐world conditions can modulate these relationships, reinforcing the need to interpret reported performance metrics within their evaluation context.

#### 3.2.2 Near‐Infrared Spectroscopy (NIR)

NIR spectroscopy is among the most mature technologies for real‐time milk composition analysis, with strong performance reported for fat and protein prediction. However, critical evaluation reveals significant limitations. Model performance is highly sensitive to calibration robustness, temperature variation, and instrument‐specific factors (Surkova et al. 2019; Díaz‐Olivares, Gote, et al. 2024; Díaz‐Olivares, Grauwels, et al. 2024). Importantly, many studies report high *R* ^2^ values under controlled conditions, but generalizability across herds, breeds, and environmental conditions remains uncertain. This discrepancy highlights a broader issue in the literature: the overreliance on laboratory validation without sufficient field‐scale verification. As a result, while NIR is technically promising, its reliability in heterogeneous production systems is still conditional.

#### 3.2.3 Biosensors for Health and Functional Markers

Biosensor technologies, particularly for mastitis detection, offer rapid and potentially cost‐effective alternatives to conventional diagnostics. Indicators such as somatic cell count, electrical conductivity, and enzyme activity are widely used (Norberg et al. 2004; Martins et al. 2019; Pan et al. 2025). However, these systems are often limited by low specificity, susceptibility to environmental variation, and maintenance challenges. Single‐sensor approaches frequently produce false positives, necessitating multi‐sensor integration and machine learning‐based interpretation. While such integration improves predictive performance, it also increases system complexity and reliance on high‐quality data. Consequently, the practical value of biosensors depends not only on detection accuracy but also on robustness, usability, and cost‐effectiveness in real‐world conditions.

#### 3.2.4 AI for Milk Quality Assessment

AI enhances milk quality assessment by enabling pattern recognition across complex datasets, including spectral, microbiological, and sensor‐derived data. Studies report high classification accuracy for adulteration detection and quality prediction (Chaudhary et al. 2022; Aqeel et al. 2025). Nevertheless, these results must be interpreted cautiously. High performance is typically achieved under controlled datasets, whereas model robustness under field variability remains underexplored. Furthermore, the integration of AI into routine workflows is often overlooked. Without alignment between prediction outputs and operational decisions, AI risks functioning as an analytical tool rather than a transformative system component. Table 1 summarizes representative performance metrics for AI‐enabled applications in dairy systems, highlighting differences between laboratory‐based and real‐world performance.

**TABLE 1:** Summary of representative performance metrics for AI‐enabled applications in dairy systems, highlighting differences between laboratory‐based and real‐world performance.

Application area	Technology/model	Reported performance	Evidence setting	Critical interpretation	Key references
Milk quality classification	KNN, Decision Tree, Random Forest, XGBoost	Accuracy ≈86%–99.5%; *R* ^2^ ≈ 0.93–0.96	Controlled datasets	High accuracy, but limited validation across diverse farm conditions	Shahzad et al. (2025), Çelik (2022)
Milk adulteration detection	FTIR + MLP, BRNN, XGBoost	Accuracy ≈97%	Laboratory datasets	Strong detection capability; robustness under real sampling variability unclear	Chu et al. (2024)
Water adulteration detection	Portable NIR + KNN, RF, SVM, SVR	Accuracy ≈99%; *R* ^2^ ≈ 0.999	Controlled experiments	Excellent analytical performance; potential overestimation due to controlled conditions	Liang et al. (2025)
Formaldehyde adulteration	UV–Vis–NIR + SVM, PLS‐DA, RF	Accuracy up to 100%; *R* ^2^ ≈ 0.998–0.999	Laboratory datasets	High sensitivity; requires external validation in real supply chains	Rosa et al. (2025)
Hyperspectral milk assessment	Hyperspectral imaging + RF, XGBoost, deep learning	Accuracy > 95%	Controlled imaging datasets	Noninvasive and rapid; cost and calibration limit routine adoption	Aqeel et al. (2025), Martinelli et al. (2026)
Milk source identification	Electronic nose + SVM	Accuracy ≈95%	Experimental dataset	Promising for traceability; sensitive to environmental and feed variations	Mu et al. (2020)
Mastitis/udder health detection	SCC, EC + ML models	Improved classification vs. single indicators	Farm‐based (limited datasets)	Multi‐sensor improves detection; false positives remain a limitation	Norberg et al. (2004), Martins et al. (2019), Pan et al. (2025)
Membrane fouling prediction	ANN, ANFIS, SVM, GA‐based optimization	Accurate fouling prediction reported	Pilot‐scale/nondairy systems	Strong predictive capability; limited real‐time industrial integration	Niu et al. (2022), Rahimzadeh et al. (2016), Rahmanian et al. (2011), Soleimani et al. (2013)
Digital twins	Hybrid simulation + AI models	Conceptual/prototype‐level performance	Limited dairy‐specific validation	High potential: insufficient industrial evidence to support transformation claims	Zhang et al. (2023), Abdurrahman and Ferrari (2025)

#### 3.2.5 Next‐Generation Dairy Processing Technologies

Emerging processing technologies, including high‐pressure processing, pulsed electric fields, and membrane filtration, play a central role in next‐generation dairy systems (Table 2).

**TABLE 2:** Processing technologies applied in dairy.

Technology	Definition	Application in next‐generation dairy processing
High‐Pressure Processing (HPP)	A nonthermal preservation method that uses very high hydrostatic pressure to inactivate microorganisms.	HHP applied at 400–600 MPa reduces pathogens ( *E. coli* , *Listeria*) in milk by 5–7 log cycles (Serna‐Hernández et al. 2021).
Pulsed Electric Field (PEF) processing	A nonthermal technique in which short high‐voltage electric pulses permeabilize microbial cell membranes. This causes microbial inactivation.	HI‐PEF at 35.5 kV/cm (1000 μs) kept whole milk microbiologically stable for 5 days at 4°C while preserving acidity, pH, lipids, and most whey proteins, with no proteolysis or lipolysis (Odriozola‐Serrano et al. 2006).
Membrane filtration (micro + ultrafiltration)	Physical separation using semi‐permeable membranes to remove bacteria and separate proteins on particle and molecular size.	Using 0.10‐μm microfiltration at 50°C, the process produced a casein‐free permeate with the highest whey protein transmission (Jørgensen et al. 2016).
Cold plasma processing	A nonthermal surface and liquid treatment using reactive ions, electrons, and radicals to inactivate microorganisms.	Dielectric barrier discharge cold plasma achieved 99.9% microbial reduction in camel milk under optimized conditions (Keewan et al. 2026).
Ohmic heating (electrical resistance heating)	A thermal process in which food is heated internally and rapidly by passing an electric current through it	Ohmic heating at 8.33–5.83 V/cm pasteurized sheep milk with 72%–73% lower energy use than conventional heating, and ≥ 4.2‐log bacterial reduction (Balthazar et al. 2022).
Ultrasound‐assisted processing	High‐intensity ultrasonic waves to enhance microbial inactivation, mass transfer, and process efficiency.	Ultrasound (20 kHz, 500 W, 3 min) on pasteurized bovine milk reduced particle size (~1 μm) and caused lower denaturation of α‐lactalbumin and κ‐casein compared with the control (Jo et al. 2019).

#### 3.2.6 Advanced Membrane Processes

Membrane technologies are central to modern dairy processing, enabling selective fractionation and improved resource efficiency (Kumar et al. 2013; Reig et al. 2021). Their integration into hybrid systems reflects a shift toward flexible and circular processing approaches. However, sustainability benefits are contingent on effective fouling management and cleaning efficiency. Without addressing these factors, energy and water savings may be offset by operational inefficiencies. This highlights the need for holistic evaluation, rather than focusing solely on technological capability.

#### 3.2.7 AI/ML‐Driven Process Optimization

AI‐based models have been applied to predict membrane fouling and optimize processing parameters, showing strong potential for improving efficiency (Niu et al. 2022). Optimization algorithms can balance throughput, energy consumption, and cleaning cycles. Despite promising results, the main limitation lies in implementation feasibility. Many dairy plants lack the infrastructure required for real‐time control adjustments based on model outputs. Therefore, the impact of AI is constrained by the degree of integration between prediction systems and operational control.

#### 3.2.8 Digital Twins and Smart Process Control

Digital twins represent a conceptual advancement in process control, enabling simulation and predictive optimization. However, current applications remain largely experimental, with limited evidence of sustained industrial deployment. The effectiveness of digital twins depends on model fidelity, sensor coverage, and validation frameworks. Without these, their role remains supportive rather than transformative. Thus, claims of their “game‐changing” potential should be considered premature.

#### 3.2.9 Advanced Dairy Ingredients and Functionalization

Advances in fractionation and processing have enabled the development of high‐value dairy ingredients, including bioactive peptides and specialized protein fractions. These innovations align with trends toward personalized nutrition and functional foods. However, the translation of bioactivity from in vitro studies to human health outcomes remains uncertain. Many studies lack clinical validation, dose–response analysis, and reproducibility across matrices. As a result, claims regarding health benefits should be interpreted with caution, particularly in the context of AI‐driven formulation strategies.

### 3.3 Circular Processing and Sustainability Considerations

#### 3.3.1 By‐Product Valorization

The valorization of whey and other by‐products is widely recognized as a key component of circular dairy systems (Ryan and Walsh 2016). Technologies such as membrane filtration and fermentation enable resource recovery and value creation. Nevertheless, economic viability and system integration remain critical challenges. Many valorization strategies are technically feasible but depend on market demand, infrastructure, and scale. This limits their widespread adoption (Rugji et al. 2025).

#### 3.3.2 Environmental Impact and LCA

Life‐cycle assessment studies highlight multiple environmental hotspots in dairy production and processing (Cruz‐Rivero et al. 2025). While mitigation strategies exist, their effectiveness varies significantly depending on system boundaries and regional conditions. This variability underscores the importance of context‐specific analysis, rather than generalized sustainability claims. Digital technologies may support monitoring and optimization, but their impact is mediated by broader system dynamics. The sustainability implications of AI‐enabled dairy systems should be evaluated using integrated environmental, economic, and social performance indicators rather than generalized assumptions regarding digital efficiency. Precision livestock farming, smart sensing, AI‐assisted optimization, and digital traceability systems may contribute to reductions in energy consumption, water use, greenhouse‐gas emissions, production losses, and operational inefficiencies; however, reported benefits remain highly context‐dependent and frequently lack long‐term field validation (Lovarelli et al. 2020). Life‐cycle assessment studies indicate that environmental hotspots in dairy production vary substantially according to system boundaries, feeding strategies, processing intensity, infrastructure, and regional production conditions (Cruz‐Rivero et al. 2025). Furthermore, sustainability outcomes are strongly influenced by economic feasibility, technology accessibility, maintenance requirements, and organizational readiness. Circular processing approaches, including whey valorization, membrane fractionation, and side‐stream biorefinery systems, may improve resource utilization and support circular bioeconomy integration, yet their scalability remains constrained by market demand, infrastructure costs, and operational complexity (Ryan and Walsh 2016; Rugji et al. 2025). Therefore, future evaluations of Dairy 4.0 systems should combine environmental indicators with techno‐economic analysis, scalability assessment, and social sustainability metrics to avoid overestimating the sustainability impact of digital technologies.

### 3.4 From Pilot to Practice: Barriers and Enabling Conditions

The transition to next‐generation dairy systems is constrained by socio‐technical factors, including regulation, infrastructure, and workforce capabilities. Data governance, interoperability, and standardization are particularly critical for scaling digital solutions. Economic barriers further limit adoption, especially for small and medium‐sized operations. Without inclusive models, digitalization risks reinforcing existing inequalities within the sector. Therefore, transformation depends not only on technological innovation but also on institutional and organizational change. Despite substantial progress in AI‐assisted sensing, monitoring, and optimization, practical implementation at industrial scale remains limited by economic, infrastructural, and operational barriers. Many AI‐enabled dairy technologies demonstrate strong predictive performance under laboratory or pilot‐scale conditions but lack validation across heterogeneous production systems characterized by variability in herd composition, environmental conditions, management practices, and processing infrastructure. Additionally, the implementation of real‐time digital systems requires substantial investment in sensor infrastructure, cloud integration, interoperability platforms, cybersecurity, workforce training, and maintenance capacity, creating important barriers for small and medium‐sized dairy operations (Malik et al. 2024a). Optimization and traceability frameworks may improve supply‐chain efficiency and transparency, yet their effectiveness depends heavily on reliable data acquisition, cross‐platform compatibility, stakeholder coordination, and governance mechanisms (Malik et al. 2022; Hastig and Sodhi 2020). Consequently, scalability should not be evaluated solely according to technical accuracy, but also according to cost feasibility, return on investment, institutional support, operational robustness, and long‐term adoption potential. These limitations suggest that the transition from experimental validation to real‐world implementation remains one of the most critical unresolved challenges in next‐generation dairy systems. Beyond technical and economic considerations, AI‐enabled dairy systems also raise important ethical and animal‐centered concerns. Increasing reliance on automated monitoring, predictive analytics, and sensor‐based surveillance may reshape the relationship between farmers, animals, and decision‐making systems. Recent studies argue that dairy technologies should not reduce animals to passive data‐generating units but should instead support welfare‐sensitive and ethically responsible management practices (Kramer and Bovenkerk 2024). While digital monitoring may improve early disease detection, feeding precision, and welfare assessment, excessive automation may also reduce direct human–animal interaction and shift decision authority toward algorithmic systems. Therefore, responsible implementation of Dairy 4.0 technologies requires transparent governance, accountability, farmer autonomy, animal welfare protection, and careful consideration of the ethical implications associated with continuous digital surveillance and automated intervention systems.

## 4 Conclusion

AI‐enabled technologies have significant potential to enhance dairy systems, particularly in sensing, quality assessment, and process optimization. However, the evidence suggests that their impact is conditional rather than inherently transformative. While technical capabilities are well demonstrated, limitations related to generalizability, scalability, and integration constrain real‐world impact. The most meaningful improvements occur when AI is embedded within sensor‐rich, interoperable systems that directly influence decision‐making processes. Thus, AI should be understood as a system‐level enabler, whose effectiveness depends on complementary infrastructure, governance, and human capacity. Rather than a universal “game changer,” AI represents a context‐dependent tool, capable of driving transformation only under specific conditions. Future research should therefore prioritize field validation, economic analysis, and integration frameworks to bridge the gap between technological potential and practical implementation.

## Author Contributions

**Zeki Erol:** conceptualization, investigation, writing – original draft, methodology, visualization, writing – review and editing, software, project administration, supervision. **Henna Hamadani:** writing – original draft, methodology, visualization, writing – review and editing, software. **Jerina Rugji:** conceptualization, investigation, writing – original draft, methodology, visualization, writing – review and editing, software, project administration, supervision. **Ambreen Hamadani:** writing – original draft, methodology, visualization, writing – review and editing, software.

## Funding

The authors have nothing to report.

## Disclosure

Statement on the use of generative AI and AI‐assisted technologies: The authors affirm that no generative artificial intelligence tools were used in the preparation of this manuscript. However, nongenerative AI tools including spelling and grammar checkers integrated in Office 365 and Google Docs, along with citation management software, were employed. All outputs from these tools were thoroughly reviewed and verified by the authors.

## Conflicts of Interest

The authors declare no conflicts of interest.

## Data Availability

The data that support the findings of this study are available on request from the corresponding author. The data are not publicly available due to privacy or ethical restrictions.
